# Nh3D: A reference dataset of non-homologous protein structures

**DOI:** 10.1186/1472-6807-5-12

**Published:** 2005-07-12

**Authors:** B Thiruv, G Quon, SA Saldanha, B Steipe

**Affiliations:** 1 Department of Biochemistry, University of Toronto, 1 Kings College Circle, Toronto, Ontario M5S 1A8, Canada

## Abstract

**Background:**

The statistical analysis of protein structures requires datasets in which structural features can be considered independently distributed, i.e. not related through common ancestry, and that fulfil minimal requirements regarding the experimental quality of the structures it contains. However, non-redundant datasets based on sequence similarity invariably contain distantly related homologues. Here we provide a reference dataset of non-homologous protein domains, assuming that structural dissimilarity at the topology level is incompatible with recognizable common ancestry. The dataset is based on domains at the Topology level of the CATH database which hierarchically classifies all protein structures. It contains the best refined representatives of each Topology level, validates structural dissimilarity and removes internally duplicated fragments. The compilation of Nh3D is fully scripted.

**Results:**

The current Nh3D list contains 570 domains with a total of 90780 residues. It covers more than 70% of folds at the Topology level of the CATH database and represents more than 90% of the structures in the PDB that have been classified by CATH. We observe that even though all protein pairs are structurally dissimilar, some pairwise sequence identities after global alignment are greater than 30%.

**Conclusion:**

Nh3D is freely available as a reference dataset for the statistical analysis of sequence and structure features of proteins in the PDB. Regularly updated versions of Nh3D and the corresponding PDB-formatted coordinate sets are accessible from our Web site .

## Background

The number of structures in the Protein Data Bank (PDB) [1] has grown to over 30,000. Many of the proteins in the PDB are homologous, i.e. have descended from a common ancestor, conserving significant aspects of their structure, function, and sequence. For purposes such as a statistical analysis of protein structure features, a subset of the PDB is required in which structural features can be presumed to be independently distributed, i.e. unbiased with respect to evolutionary descent.

A number of PDB subsets have been proposed to address this need. The most often used subset of structures in the PDB is the PDBSELECT [2,3]. This provides lists of chains compiled at predefined maximum percent sequence identities. The most stringent cutoff employed is a maximum of 25% residue identity between chains. Other subsets culled at predefined sequence identity are available from the PDB itself. In recent years, servers such as the PDB-REPRDB [4,5] and PISCES [6] allow users to compile customized lists of protein chains based on structure quality and maximum mutual sequence identities. PISCES allows the user to define additional restriction parameters such as the minimum sequence length and maximum R-value. However, all of the above-mentioned lists use sequence similarity as the defining criterion for protein selection and include distantly related but recognizably homologous proteins.

In order to define a reference dataset of non-homologous protein structures, we have constructed a tool to extract coordinate subsets from the PDB, driven by CATH [7] domain topology information. The underlying hypothesis is that differences in the topology of protein secondary structure arrangements are incompatible with the notion of gradual, divergent evolution from a common ancestor. The experimentally best-defined representatives of domain sets at the CATH Topology level are selected, the structures are validated with respect to sequence/structure similarity that might be indicative of homology, and homologous internal repeats are purged to remove redundancies. Our reference database of non-homologous, dissimilar structures is versioned and automatically updated with every revision of the CATH database.

## Construction and contents

The CATH database is a hierarchic classification of protein domains. At the Topology level of the hierarchy (similar to the Fold-level of SCOP [8]), the domains are globally dissimilar and thus provide a set of structures that are not recognizably homologous.

**Figure 1:**
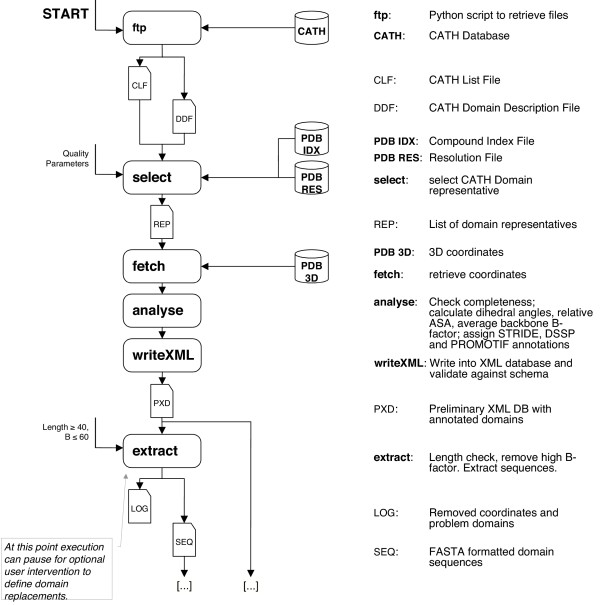
**Data Flow Diagram for Nh3D, from start to a list of domain sequences. **For details see text.

To create Nh3D (Figure 1), representative structures were selected for each of the Topology for classes 1–4 in the CATH hierarchy (Version 2.5.1: released January 2004). We have not considered proteins classified into classes 5–9, because these assignments are preliminary. Using the PDB resolution and compound index files, we have selected the best representative for each Topology, that minimally fulfill the following criteria:

1) X-ray or neutron-diffraction structure, resolved at 2.2 Å or better (since we consider NMR structures to be less reliable for the statistical analysis of detailed structural relationships);

2) Domain has a minimum length of 50 residues (since we consider shorter structured domains, frequently dominated by a large percentage of disulfide bonds, to be atypical for independent folding units);

3) Engineered structures are excluded, unless no alternatives are available (we exclude structures that contain the sub-strings {MUTAT | MUTAN | REPLACE} in their description line of the compound index file since engineering may have introduced structural strain that has not been resolved through evolutionary optimization of the protein.).

When two or more structures meet the selection criteria, the one with the better resolution is chosen as the representative. For structures that have the same resolution, the one with the better Ramachandran Z score (obtained from the PDBFINDERII [9] database) is chosen as the representative.

**Figure 2:**
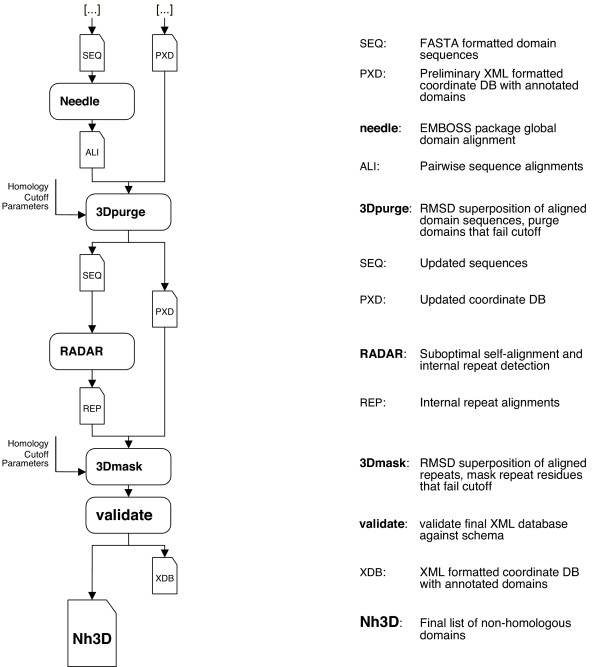
**Data Flow Diagram for Nh3D, from a list of domain sequences to the final reference dataset. **For details see text.

To ensure that these structurally dissimilar domains are indeed not recognizably homologous, two stages of validation and purification are employed (Figure 2).

### 1) Evaluation of structural and sequence similarity between the domains

We check for sequence similarities that correspond to globally similar structures, to guard against the inclusion of homologous subdomains that might be variably assigned to different Topologies due to imprecise definitions of structural domains. Exhaustive pairwise global alignments of all domains are performed using the Needle program from the EMBOSS suite [10] with the EBLOSUM62 similarity matrix and default gap and extension parameters of -10.0 and -0.5. The RMSD of aligned residues after optimal superposition between their backbone atoms is calculated by the Kabsch method [11]. Domains with greater than 40 residues aligned (a conservative limit for foldable and thus independently inheritable domains), possessing greater the 25% sequence identity (a threshold commonly used to confidently identify homologues through sequence analysis) and superimposable at less then 3.8 Å RMSD (the average Cα – Cα distance that defines the limits of a meaningful pairwise residue superposition) are considered homologous. The smaller of the two domains is removed from the dataset. None of the domain pairs in this version of the CATH database met the above criteria for exclusion; this emphasizes the reliability of CATH domain assignments.

### 2) Identification of repeat regions in domains

Internal repeats may arise due to internal duplication events and are known to occur more frequently after the first duplication [12]. The internal repeats in Nh3D are identified using the program RADAR [13]. The RMSD after optimal superposition between the RADAR aligned repeat residues is calculated by the Kabsch method. Repeats with greater than 25% sequence identity and less than 3.8Å RMSD are considered redundant and removed from the database.

We annotate the final set of protein structures with the following information:

a) secondary structure assignment using DSSP [14] and STRIDE [15] algorithms

b) relative side-chain solvent accessible surface areas using the Shrake and Rupley [16] approach and a reference dataset of modeled, extended GLY-XXX-GLY tripeptides

c) regions with high B-Factor (where the average B-factor of the backbone atoms of three consecutive residues is found to be greater than 60 Å ^2^are identified and flagged)

d) beta and gamma turn assignments using PROMOTIF [17]

Residues in the PDB coordinate files that have missing backbone residues are identified and removed from the dataset. Modified residues that appear as 'HETATM' records in the PDB files are treated as chain breaks; a distance check between bonded backbone atoms is implemented to check for additional discontinuities in a chain.

Nh3D is freely available in an easily parseable, fully specified flatfile format and as PDB-formatted coordinates. Differently formatted output for specific needs can be made available upon request to the authors.

## Utility and discussion

Our dataset uses coordinates from 477 proteins consisting of 570 of 820 CATH Topologies and contains 90780 residues. The chosen toplogies represent over 90% of the structures in the PDB classified by CATH version 2.5.1.

Interestingly, we observe numerous domains with striking sequence similarity (> 30 % over the aligned residues), with completely dissimilar structure. A single likely homologue (CATH domains 1d0cA3 and 1nos03, 42.7 % identity in 89 aligned residues) has been identified in CATH Version 2.5.1. However, the domain 1nos03 was already excluded from the database in a previous step, due to the high residue B-factors over the length of its domain.

Significant internal repeats were removed from 38 domains. For example, repeats were found in representatives of Porin, Tachylectin-2, and MutS topologies. Other repeats, most notably those of TIM barrel proteins, did not meet our cutoff criteria for recognizable homology and hence were retained.

A comparison of our results with a sequence based PDB subset (PDBSELECT of October 2004) shows the large number of redundant, similar structures that cannot be eliminated based on sequence dissimilarity alone. The most redundant is the Rossman fold (CATH: 3.40.50, 105 families; Nh3D representative: 1GCI:1-275; PDBselect: 138 sequences), the second most redundant are immunoglobulin-like domains (CATH: 2.60.40, 52 families; Nh3D representative: 1OE1:A:2-152; PDBselect: 93 sequences). While it has been a matter of discussion whether such "superfolds" might be the result of convergent evolution [see e.g. [18]], we take a conservative approach with Nh3D. In constructing the reference dataset by selecting a single representative for every CATH Topology, the residual chance of inappropriately including homologues is minimized. Nh3D does not attempt to be exhaustive at this point. For example, our "immunoglobulin-like" topology representative is the human copper, zinc superoxide dismutase, 1MFM:A:1-153, however the metal-binding greek-key proteins and the immunoglobulin superfamily, both of which are included in this class, may indeed be examples of convergent evolution towards a common fold. Since Nh3D is a representative sample of detailed conformations in protein structures, completeness is not an issue, given that the number of excluded analogous folds is small relative to those folds for which we do not yet possess high-resolution structures. Nevertheless, we wish to emphasize a continuing need to further improve on the automated definition and classification of dissimilar folds.

## Conclusion

Nh3D is freely accessible as a reference dataset for the unbiased statistical analysis of sequence and structure features of proteins in the PDB. While our own motivation was to construct a source dataset for an analysis of short peptide conformations, we anticipate many uses of Nh3D, including the calibration of algorithms for the detection of distant gene relationships, for the recognition of false positives in sequence or structure alignments, or for the prediction of protein structure.

## Availability and requirements

The dataset, a list of best representatives for CATH Topologies that did not meet our criteria, documentation, format specifications and supplementary information can be downloaded from . Nh3D Version 2.0 based on CATH 2.6.0 has since been made available.

## List of abbreviations

CATH-A hierarchical classification of protein domain structures

DSSP-Definition of Secondary Structure of Proteins

EMBOSS – European Molecular Biology Open Software Suite

NMR-Nuclear Magnetic Resonance

SCOP-Structural Classification of Proteins

STRIDE – STRuctural IDEntification method

PDB-Protein Data Bank

RADAR – Rapid Automatic Detection and Alignment of Repeats

RMSD – Root Mean Square Deviation

WWW-World Wide Web

## Authors' contributions

BTG wrote the second version of the code to generate Nh3D and helped to draft the manuscript.

GQ wrote the first version of the code and helped to prepare the manuscript.

SAS participated in the design and helped to draft the manuscript.

BS conceived of the study, and participated in its design and coordination and drafted the manuscript.
